# RNAi technology development for weed control: all smoke and no fire?

**DOI:** 10.1002/ps.8729

**Published:** 2025-02-21

**Authors:** Silvia Panozzo, Andrea Milani, Serena Bordignon, Laura Scarabel, Serena Varotto

**Affiliations:** ^1^ Institute for Sustainable Plant Protection (IPSP), National Research Council of Italy (CNR) Legnaro Italy; ^2^ Department of Agronomy Food Natural Resources Animals and Environment (DAFNAE), University of Padova Legnaro Italy; ^3^ Present address: Belgian Nuclear Research Centre (SCK•CEN) Mol Belgium; ^4^ Present address: Department of Biotechnology, Faculty of Bioscience Engineering Ghent University Ghent Belgium

**Keywords:** RNA interference, dsRNA, weed control, gene silencing target, model weed species

## Abstract

RNA interference (RNAi) technology, specifically Spray‐Induced Gene Silencing (SIGS), holds potential as an innovative approach for selective weed control, promising environmentally friendly alternatives to traditional herbicides. Although the development of RNAi‐based crop protection agents against pathogens, insects and viruses is advancing rapidly, RNAi‐based weed control remains in the nascent stages, with challenges in gene target specificity and effective delivery mechanisms. It is potentially a game‐changer in agriculture, yet SIGS's applicability is limited by the lack of scientific evidence. The overall aim of this review is to focus attention on critical points that need to be addressed to advance the knowledge about and development of RNAi herbicides, and overcome the poor progress achieved so far. Enhancing RNAi delivery methods and focusing on high impact weed species could transform SIGS into a viable tool for sustainable agriculture. © 2025 The Author(s). *Pest Management Science* published by John Wiley & Sons Ltd on behalf of Society of Chemical Industry.

## Graphical abstract

Despite the potential of Spray‐Induced Gene Silencing in weed management several critical points such as molecular target, weed species of interest as well as delivery techniques need to be addressed.

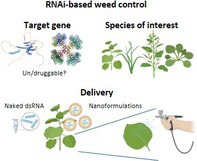

## 1 INTRODUCTION

Understanding how foliar‐applied RNA induces RNAi (RNA interference) in plants might result in the development of novel biotechnological approaches with a unique mode‐of‐action (MoA) compared to all of the commercial products currently used for crop protection.^1^, ^2^ Therefore, this strategy could represent an alternative, or integrative, tool to conventional weed management strategies, commonly based on chemical products acting on specific target enzymes. The need to develop innovative crop protection technologies ensuring high weed control (equivalent to conventional herbicides) and crop yield is becoming more evident every day, because chemical weed control is facing very relevant difficulties: (i) the increasing regulation on the use of agrochemicals owing to environmental and health concerns; (ii) the lack of new sites of action and molecules on the market and increasing registration costs; and (iii) the continuous evolution of herbicide‐resistant weeds,^3^ lowering the effectiveness of the most used (and safer) molecules and representing a potential waste of ineffective herbicide (also causing unwanted environmental pollution) and a huge loss of profit.

Spray‐Induced Gene Silencing (SIGS) has been proposed as a next‐generation weed management method.^4^ The basic principle is spraying target weeds with RNA molecules able to activate the plant RNAi mechanism and induce post‐transcriptional gene silencing (PTGS). Although numerous studies have confirmed the feasibility of exogenously applied double stranded RNAs (dsRNAs) to protect plants against pathogenic viruses,^5^ fungi,^6^ insects,^7^ mites and nematodes, published peer‐review studies on the silencing of endogenous genes to protect crops against weed competition are lacking. Successful silencing of endogenous genes after topical application of dsRNAs was achieved at the laboratory‐level exclusively for gene functional studies in *Dendrobium hybrid* (an orchid),^8^ *Vitis vinifera* (grape)^9^ and in the model species *Arabidopsis thaliana* and *Nicotiana benthamiana*.^10^, ^11^ To date, the activity of herbicidal RNAi has been tested in a few weeds, with variable results. As two examples, plants of the aquatic weed *Mikania micrantha* were repeatedly treated for 14 days with dsRNAs before observing a phenotypic effect,^12^ and systemic silencing of the magnesium chelatase gene was obtained in *Amaranthus cruentus*.^11^ One of the first reports of RNAi with herbicidal activity refers to a patent by Monsanto (US20110296556A1, 2011), targeting *Amaranthus palmeri* and *Kochia scoparia*, while in 2012 the company announced the RNAi‐based technology platform called BioDirect™, with both insecticides and herbicides being developed. Despite the promotion and the very suggestive premises of SIGS, no commercial product reached the market until this year. The first RNAi pesticide (Calantha™, active ingredient ledprona, CAS# 2433753‐68‐3), an insecticide developed to specifically control Colorado potato beetle (*Leptinotarsa decemlineata*), was approved by the U.S. Environmental Protection Agency (EPA) in 2024. The approval of such a product represents a real milestone in crop protection and will hopefully pave the way for other RNAi pesticides, including herbicides.

The authors of a recent opinion paper about herbicidal SIGS highlighted that the main constraints to its development are (a) the stability and delivery of RNA molecules inside plants, (b) identifying targetable sequences specific for weeds and harmless for crops, (c) the availability of sequenced and annotated genomes for weeds, and (d) RNA molecule synthesis costs.^2^ In this review we suggest possible hints to explore the feasibility of herbicidal SIGS by focusing first on some weeds with specific characteristics, such as their demonstrated capability of evolving resistance to chemical herbicides, and the availability of genomic resources and reverse genetic tools. We also discuss the advances in dsRNA delivery mediated by nanoparticles (NPs), specifically focusing on weeds, aiming to give useful hints to researchers interested in this topic and give some insights into the synthesis costs of RNA molecules.

### 1.1 RNA interference in plants: molecular mechanism and main developed methods

**Figure 1:**
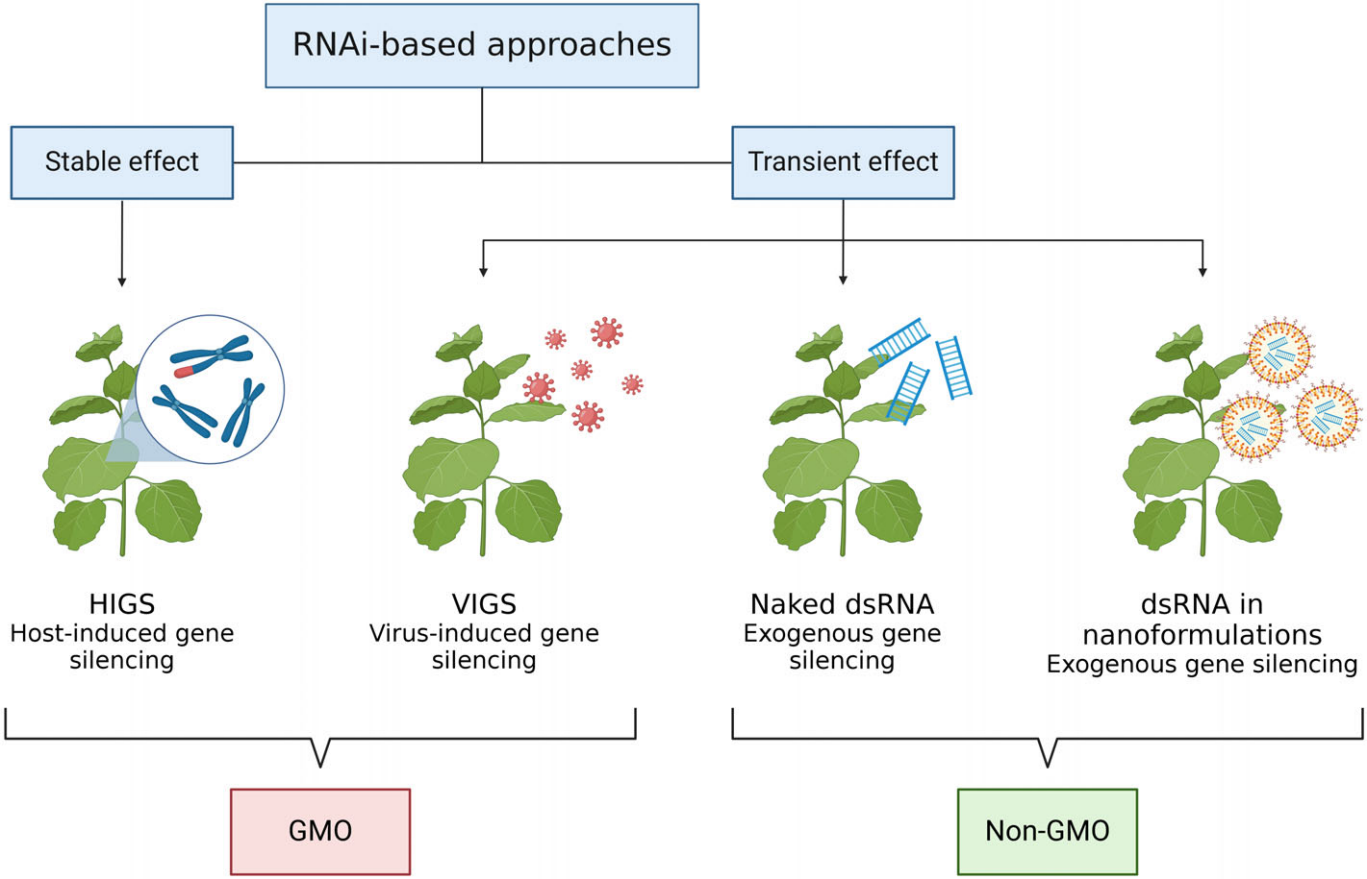
RNA interference applications in plants. To obtain a stable effect, plants must be genetically modified, while a transient effect is obtained by applying modified viral plasmids (virus‐induced gene silencing, VIGS) or dsRNAs. HIGS, host‐induced gene silencing, is a technology where GMO crops express dsRNAs with pesticidal activity. Created in biorender. Bordignon, S. (2024) https://BioRender.com/d79v637.

RNA interference is a critical natural regulatory and defense mechanism in plants, mediating the control of growth, development, and responses to pathogens and environmental stresses through gene silencing by small RNAs. DICER‐like proteins (DCLs) process double‐stranded RNA (dsRNA), derived from sources such as viruses, transposons, or endogenous transcripts, into small interfering RNAs (siRNAs). These siRNAs bind to ARGONAUTE (AGO) proteins to form effector complexes. Different DCLs are engaged based on the dsRNA origin, producing small RNAs with distinct sizes and functional roles. The biogenesis and characteristics of these small RNAs influence whether post‐transcriptional gene silencing (PTGS), RNA‐directed DNA methylation (RdDM) or transcriptional gene silencing (TGS) is activated (see ref. 13 for a more detailed description of these mechanisms).^13^ For our discussion, PTGS is the most relevant mechanism, because it causes the degradation of target mRNA and thus the silencing of the relative genes. RNAi has been widely used in plants in reverse genetics and functional genomics research and is preferred over mutants, when mutations are associated with gene knockdown and lethal phenotypes.^14^ In general, RNA interference‐mediated silencing can be stable or transient (Fig. 1): to obtain a stable RNAi effect, plants are genetically modified, whereas to obtain a transient effect plants are topically treated with dsRNAs or viral plasmids (i.e. virus‐induced gene silencing, VIGS). In crop protection, crops can be genetically modified to express RNA molecules with pesticidal activity; one example is the 240‐bp dsRNA fragment of the *Snf7* gene of *Diabrotica virgifera virgifera* that is integrated into the MON87411 maize line developed by Monsanto.^15^ This is called host‐induced gene silencing (HIGS). The use of this technology is quite intuitive for the control of insects and pathogens, but not applicable for weed control, except for parasitic weeds.^16^ Instead, in VIGS, the plants are treated with viruses engineered to express dsRNA targeting an endogenous gene. Both methods are widely used at the laboratory scale for functional studies, yet in countries where the development and/or the use of engineered organisms is not allowed, only the spray application (most likely foliar) of RNA (i.e. SIGS) would be transferable to the field. Whether it is better to use long or short RNAs to develop an RNAi herbicide would deserve a dedicated review, because the whole RNAi process is still not fully understood, and many factors should be taken into consideration. It was demonstrated that RNAi can be obtained by topically applied dsRNAs with a size of 21–24 bp or >139 bp,^17^ but it seems that only 22‐bp small RNAs spread systemically through the plant.^11^, ^18^ Furthermore, small RNAs would potentially be more specific than long dsRNAs, and therefore more suited to the development of technology, but at present the production costs are far higher with respect to long dsRNAs, because of different production methods (chemical/*in vitro versus in vivo*).^19^ To formulate an effective RNAi herbicide, considering mixing sequences with different targets^20^ or sizes, and eventually adding some specific elicitors of RNAi, cannot be excluded.

### 1.2 The untapped potential of RNAi herbicides is linked to a broadening of possible molecular targets

**Figure 2:**
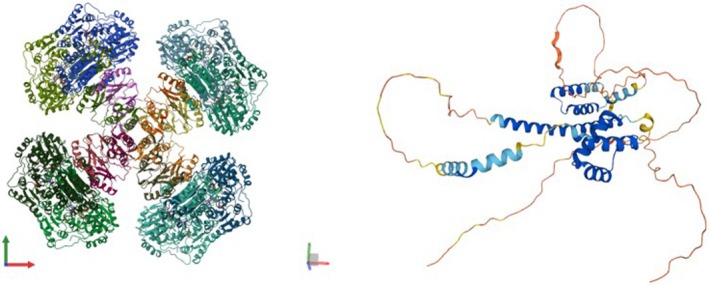
Comparison between the well‐defined structure of a typical druggable target, the enzyme acetolactate synthase (x‐ray determined, PDBe: 6u9h) and the undefined structure of an undruggable target protein, the Homeobox protein SHOOT MERISTEMLESS (AlphaFold‐predicted, Uniprot: Q38874). This protein has been successfully silenced in the parasitic plant *Cuscuta pentagona* by the small interfering RNA produced by a genetically modified host.^26^

The development of herbicides with low environmental impact is a good reason to investigate RNAi technology, but the potential of such a technology very likely lies in the broadening of possible molecular targets. Silencing the well‐known herbicide target gene/protein would be quite intuitive, yet the potential of RNAi for weed control would be completely exploited only if the so‐called undruggable targets are identified. An undruggable target is either a protein or a molecular pathway that is difficult or impossible to modulate with conventional drugs (such as herbicides).^21^ This is because undruggable targets lack binding sites for the drug. Such binding sites are normally placed on the active sites of essential enzymes (or near them) impeding the natural substrate being processed, or have unclarified 3D structures (see Fig. 2 for examples of this kind of proteins).^21^, ^22^ In mammals, examples of undruggable targets are small GTPases (guanosine triphosphate hydrolases), phosphatases, transcription factors and epigenetic factors.^21^ In plants, the list should be similar, given that among these, only serine/threonine protein phosphatases can be inhibited (by a few molecules). To provide an indication of the vast potential of silencing the undruggable targets, it has been estimated that only 15% of the 30 000 predicted human protein‐coding genes had the potential to be exploitable as drug targets,^23^ meaning that the remaining 85% of the proteome was deemed undruggable (and potential target of RNAi). Unfortunately, there is no similar estimate for plant genomes, but if we hypothesize that the proportions are somewhat similar, the number of possible RNAi targets might be very high. Indeed, having a complete atlas of the druggable and undruggable targets in the *A. thaliana* genome would be very useful for plant and weed biologists.

Although all coding sequences might be targeted by RNAi, not all are expected to be equally good candidates for a RNAi‐based weed control. For example, nuclear genes should be easier to study than chloroplast‐encoded genes, because the regulation of chloroplast gene expression by microRNA is still a very poorly understood process.^24^ In general, the molecular target (i) should be essential for plant survival or (ii) should substantially alter the fitness or reproductive/competitive ability of the plant itself, (iii) its silencing should cause extensive pleiotropic effects, and (iv) its silencing should not be complemented by redundant genes/pathways. Another critical point is that the target gene must be actively expressed at the developmental stage at which weeds are normally treated (for example 2–4‐leaf stages), in general after emergence and before crop canopy closure. Furthermore, the target gene sequence must be known or experimentally determined. Most of the genes encoding for chemical herbicide targets have these characteristics and are very well‐known. Thus, they are supposed to be good candidates for developing RNAi herbicides. However, dozens of *A. thaliana* loss‐of‐function mutants, resulting in lethal phenotypes, were discovered in the past (e.g. embryo‐defective *emb* mutants)^25^ and they might be explored as potential RNAi target genes. Because no known herbicide kills plants by completely suppressing an enzyme but rather by causing toxic metabolite accumulation owing to partial inhibition,^22^ the partial silencing of target genes also is expected to result in plant death or stunting. Indeed, for most of those genes the function and expression level at each phenotypic stage has been determined only in *A. thaliana*, whereas all of this crucial information must be experimentally determined case‐by‐case (likely, weed‐by‐weed). An example of how to select a candidate gene using the TAIR database (https://www.arabidopsis.org/) can be found in the Supporting information, Fig. S1.

### 1.3 All weeds are equal, but some weeds are more equal than others

Given that there are thousands of species that can be considered weeds, which ones should we start from? Developing foliar applied RNAi in *A. thaliana* (or other model species) might be tempting for obvious reasons, yet we believe that research on herbicidal RNAi should focus on true weeds, because (as highlighted elsewhere) *A. thaliana* lacks most of the weedy traits.^27^ *Arabidopsis thaliana* has a very small genome compared to most weeds, and therefore redundancy might be reduced and potential effects of RNAi much enhanced. Additionally, biochemical pathways and gene regulation might be very different among species. By now, new technologies (e.g. ‘omics’ disciplines) allow studies in nonmodel plant species,^28^ so there is no reason to work only on model species.

Theoretically, any weed species can be targeted by RNAi, but the technology being at the early stages of development, research should give priority to weeds having specific characteristics, at least to obtain a working proof‐of‐concept. First of all, weed species that have evolved resistance to many sites of action should have priority as they are the most difficult to control owing to the lack of effective alternatives. In Table 1, a list of economically pernicious weeds that have evolved resistance to four or more sites of action is reported.^3^ A critical point to develop dsRNAs with herbicidal activity is the availability of specific genomic resources, to have the molecular tools for the development of specific dsRNAs ensuring selectivity and avoiding off‐target silencing. From this point of view, very impressive work has been done by the International Weed Genomic Consortium (IWGC: https://www.weedgenomics.org/), that has made 29 weed genomes available on its website and has aided the sequencing of many others, and even more are in progress.^29^ Thanks to IWGC and other genomics‐oriented working groups, plus the huge decrease in sequencing costs over the last 20 years, genome information is no longer an issue.

**Table 1:** List of economically pernicious weed species that have evolved resistance to four or more sites of action (see https://weedscience.org/ for further details).^3^ For each species, the availability of a reference genome is reported in the second column. If the species genome is not available yet, the closest congeneric species with a sequenced genome is reported

Weed species	No. of sites of action involved	Is the weed (or a congeneric species) genome sequenced?
*Poa annua*	10	Yes
*Lolium rigidum*	9	Yes
*Amaranthus palmeri*	7	Yes
*Avena fatua*	7	Yes
*Eleusine indica*	7	Yes
*Echinochloa crus‐galli* var. crus‐galli	6	Yes
*Lolium perenne* ssp. *multiflorum*	6	*Lolium perenne*
*Amaranthus hybridus* (syn: *quitensis*)	5	Yes
*Amaranthus retroflexus*	5	Yes
*Amaranthus tuberculatus* (=*A. rudis*)	5	Yes
*Arctotheca calendula*	5	No
*Conyza sumatrensis*	5	Yes
*Raphanus raphanistrum*	5	Yes
*Echinochloa colona*	5	Yes
*Lolium perenne*	5	Yes
*Kochia scoparia*	4	Yes
*Ambrosia artemisiifolia*	4	Yes
*Bidens pilosa*	4	*Bidens hawaiensis*
*Conyza bonariensis*	4	Yes
*Alopecurus myosuroides*	4	Yes
*Avena sterilis* ssp. *ludoviciana*	4	*Avena fatua*
*Hordeum murinum* ssp. *glaucum*	4	*Hordeum vulgare*
*Sorghum halepense*	4	Yes

### 1.4 Tools to find an effective RNAi winning combo (weed species‐target)

Given that all the target sequences need to be validated first at the laboratory scale, applicability to the selected weed of easy, fast and effective reverse genetics tools such as VIGS is mandatory. Virus‐induced gene silencing is a flexible, efficient and high‐throughput approach, providing insights that are crucial for plant biology, breeding and biotechnology applications. This approach can be used to obtain rapid functional gene analysis, because it allows researchers to transiently modify the expression of target genes, with no need for stable transformation. VIGS can be used on many plant species, including those for which stable transformation is challenging (or economically not justifiable), making it versatile for a wide range of plants, including nonmodel organisms, as weeds are. Unfortunately, weed‐specific VIGS protocols have been developed for only a few species (Table 2).

**Table 2:** List of weed species for which an effective VIGS protocol has already been published.

Weed species with working VIGS	Dicot/monocot	Family	Virus vectors
*Alopecurus myosuroides* Huds.	Monocot	Poaceae	BSMV – FoMV^30^
*Avena fatua* L.			BSMV^31^
*Setaria viridis* (L.) P. Beauv.			FoMV^32^
*Lolium temulentum* L.			BSMV^33^
*Solanum nigrum* L.	Dicot	Solanaceae	TRV^34^
*Ipomoea purpurea* (L.) Roth		Convolvulaceae	TRV^35^
*Striga hermonthica* (Delile) Benth		Orobanchaceae	TRV^36^

BSMV, barley stripe mosaic virus; FoMV, foxtail mosaic virus; TRV, tobacco rattle virus.

Among the 23 weed species reported in Table 1, only *A. myosuroides* and *A. fatua* have published VIGS protocols (Table 2). For some other species VIGS protocol might be adapted starting from those developed for congeneric species (Table 3). The fine‐tuning of VIGS protocols to weed species also would help gene functional studies, for example to validate the role of genes suspected of conferring resistance and/or those differentially expressed between susceptible and resistant biotypes. A substantial difference between VIGS protocols developed for dicots and monocots is that dicots are normally directly infected by the *Agrobacterium tumefaciens*/virus system, whereas for monocots it is necessary to first infect an intermediate host (normally *N. benthamiana*).^30^ Given their worldwide distribution and resistance‐related issues, developing VIGS for weedy *Lolium*, *Echinochloa, Amaranthus* species and *Sorghum halepense* (L.) Pers. would surely be of great interest.

**Table 3:** List of economically pernicious weed species having VIGS protocols designed for congeneric species.

Economically pernicious weed species candidate for VIGS	Dicot/monocot	Family	Congeneric species with working VIGS	Virus vectors
*Lolium rigidum* Gaud.	Monocot	Poaceae	*Lolium temulentum* L.	BSMV^33^
*Lolium perenne* subsp. *multiflorum* (Lam.) Husnot				
*Lolium perenne* L.				
*Avena sterilis* L.			*Avena fatua* L.	BSMV^31^
*Hordeum murinum* subsp. *glaucum* (Steud.) Tzvelev			*Hordeum vulgare* L.	BSMV^37^
*Sorghum halepense* (L.) Pers.			*Sorghum bicolor* (L.) Moench.	BMV^32^
*Amaranthus palmeri* S. Watson	Dicot	Amaranthaceae	*Amaranthus tricolor* L.	TRV^38^
*Amaranthus hybridus* L.				
*Amaranthus retroflexus* L.				
*Amaranthus tuberculatus* (Moq.) J.D. Sauer				
*Conyza sumatrensis* (Retz.) E. Walker		Asteraceae	*Conyza blinii* H.Lév.	TRV^39^
*Conyza bonariensis* (L.) Cronq.				
*Raphanus raphanistrum* L.		Brassicaceae	*Raphanus sativus* L.	TYMV/TRV^40^

BSMV, barley stripe mosaic virus; BMV, brome mosaic virus; TRV, tobacco rattle virus; TYMV, turnip yellow mosaic virus.

### 1.5 RNA molecules need a Trojan horse to be effective

As mentioned before, VIGS can only serve as an experimental tool, but to make the technology applicable it is necessary to find a way to apply the RNA molecules topically. The mere foliar application of nude RNA molecules does not seem to be a successful strategy to effectively silence endogenous plant genes, given that they must cross multiple barriers to reach the cytoplasm of plant cells (where RNA interference occurs). The outermost layer of the leaf, composed of a waxy cuticle, is the first barrier. It is designed to protect against water loss and pathogen entry, and it limits the penetration of large molecules (such as dsRNAs). Beneath the cuticle is the cell wall, a rigid structure made up of cellulose, hemicellulose and pectin. It acts as a second barrier, providing structural support and additional protection against the entry of foreign particles. After passing through the cuticle and cell wall, the dsRNAs must cross the plasma membrane to enter the cell's cytoplasm. The plasma membrane is selectively permeable, and dsRNAs usually requires specific transport mechanisms to pass through it because it cannot freely diffuse across. Indeed, dsRNA molecules are negatively charged as a consequence of their phosphate backbone, making the crossing of hydrophobic (water‐repellent) barriers such as lipid membranes very difficult. Apparently, some results can be obtained by several delivery methods, such as high‐ and low‐pressure spraying, infiltration, petiole absorption, petiole or trunk injection, spreading by pipette or brushes, mechanical inoculation, root/seed soaking and soil/root drenching. Even if interesting from the research point of view, none of these methods are applicable at the field level. Instead, very interesting results were obtained by using NPs as RNA carriers. The use of nanocarriers as drug delivery systems is a very active field of research, and there is plenty of information available, but still not much about their use to increase the efficacy of RNA molecules with herbicidal activity. The first study described the silencing of two *A. thaliana* developmental genes with a cationic fluorescent NP applied to roots of 10‐days old seedlings.^41^ Six years later, single‐walled carbon nanotubes were reported to be used to deliver dsRNA molecules in *N. benthamiana*, to effectively silence the disease resistance endogenous gene ROQ1.^42^ Likewise, carbon dots charged with small RNAs (22 bp) were used in the same species to silence both the green fluorescent protein (GFP) transgene and the endogenous gene encoding the H and I subunits of magnesium chelatase (CHLH and CHLI).^43^ Again in *N. benthamiana*, DNA nanostructures were demonstrated to help in the delivery of short RNAs: using this technique, both the GFP transgene and ROQ1 were silenced after foliar infiltration.^44^ In the same species, clay NPs efficiently delivered small interfering RNA to intact plant leaf cells and resulted in silencing of the GFP transgene 1 day after the leaf infiltration.^45^ In *N. benthamiana*, mesoporous silica NPs were used to mediate siRNA delivery and achieve a long‐term silencing for several endogenous genes.^46^ Because stomatal openings are measured on a micrometer scale (≈10 × 20 μm),^47^ they are unlikely to hinder the uptake of RNA molecules (whether naked or carried by NPs) when open.^48^ Instead, crossing the cell wall and plasma membrane poses a far greater challenge, needing the adoption or development of NPs with specific chemical characteristics to overcome these barriers. More information about the issue of enhancing dsRNAs delivery to the plant cells through nanocarriers can be found in a review written by Da Silva *et al*. (although not focusing on herbicidal dsRNAs).^49^

## 2 CONCLUSIONS AND FINAL REMARKS

In conclusion, developing a working proof of concept for RNA‐based bioherbicides hinges on advancing our knowledge across three critical areas: the plant RNAi mechanism, VIGS and the use of nanocarriers for RNA delivery. A key priority is refining VIGS protocols for diverse and impactful weed species, as current methods are limited to a few model plants and may not accurately reflect the complex genetic and physiological traits of weedy species in real‐world conditions. Likewise, effective RNA delivery mechanisms are crucial for moving RNA herbicides from laboratory studies to field applications. Tailored nanocarrier technologies that can enhance RNA uptake and stability in field conditions will be crucial in transforming RNAi‐based weed control into a viable agricultural tool. Last, developing new technologies to produce cheaper small RNA is desirable. A (completely speculative) possibility that should be taken into consideration is the digestion of long dsRNAs (tandem‐repeat of proven‐effective short RNA) with Dicer‐like proteins, or Cas13 or ribozymes, specifically engineered to cut the dsRNAs in a sequence‐dependent manner, or the *in vitro* amplification of small RNA with engineered RNA‐dependent RNA polymerase (RdRp). Twenty years ago, very few would have bet on the incredible reduction of sequencing costs; perhaps even RNA manipulation technology will undergo such a far‐reaching change.

Ultimately, increased investment in these areas of research and development is necessary to overcome current limitations and harness the full potential of RNAi herbicides. By focusing on these advancements, RNA‐based approaches could emerge as a powerful, eco‐friendly alternative to chemical herbicides, supporting sustainable agriculture and addressing the urgent issue of herbicide resistance in key weed species.

## AUTHOR CONTRIBUTIONS

Conceptualization, S.P and S.V.; writing—original draft preparation, S.P., A.M. and S.B.; writing—review and editing, L.S. and S.V.; supervision, S.V; Funding acquisition, S.P. All authors have read and agreed to the published version of the manuscript.

## CONFLICT OF INTEREST

The authors declare no conflict of interest.

## Supporting information


**Data S1.** Supporting Information.

## Data Availability

Data sharing not applicable to this article as no datasets were generated or analysed during the current study.
