# Supplementary material for: RNAi technology development for weed control: all smoke and no fire?
Source: Pest Manag Sci. 2025 Feb 21;81(7):3430–6. doi: 10.1002/ps.8729 (PMC12159382; doi:10.1002/ps.8729)
Supplement: Supplementary file 1 — Data S1. Supporting Information. [file PS-81-3430-s001.docx]

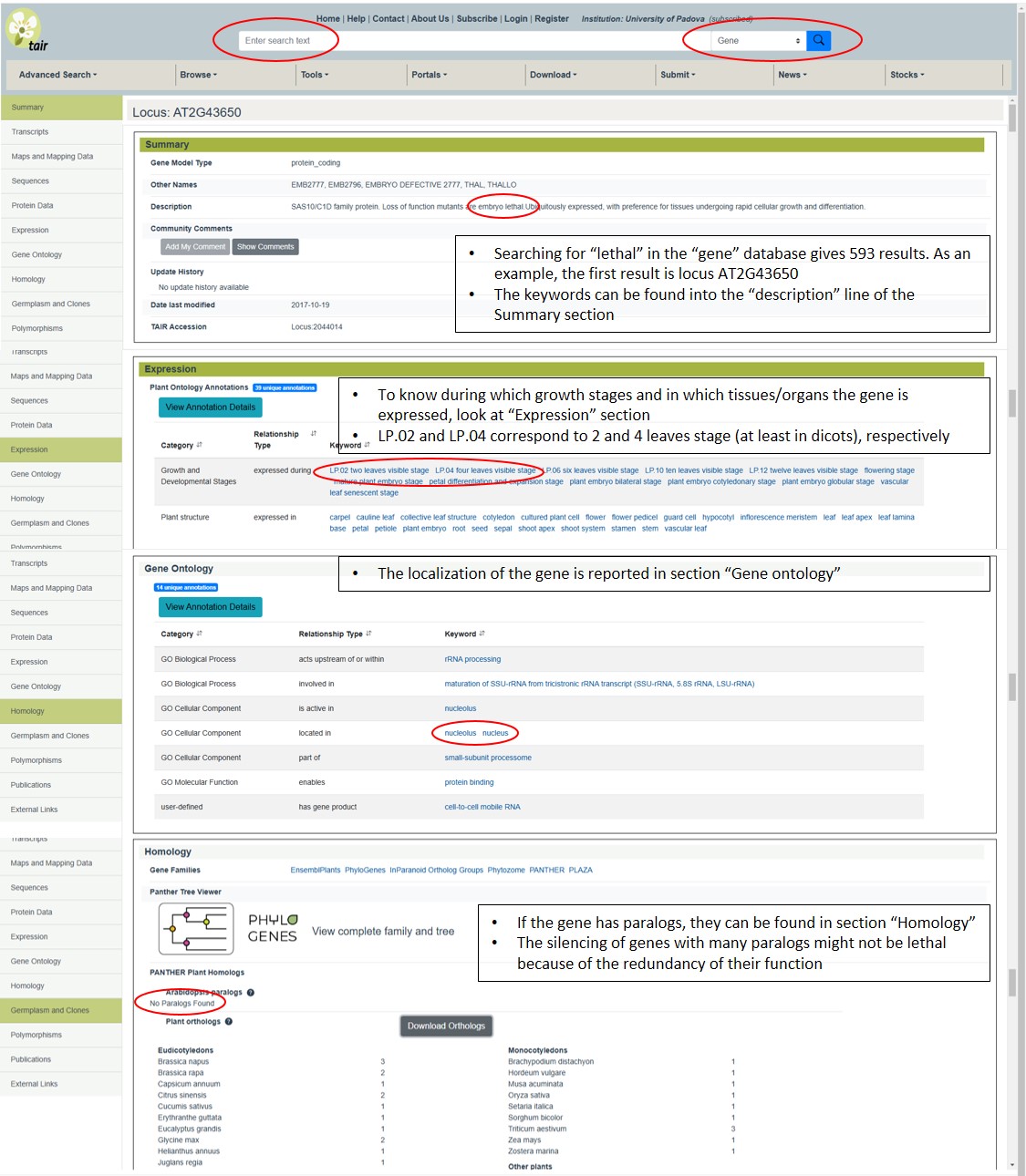


Figure S1. An example of how to select a candidate gene using the TAIR database (https://www.arabidopsis.org/)
